# Foreign Correspondence

**Published:** 1888-09

**Authors:** 


### CORRESPONDENCE.

### FOREIGN CORRESPONDENCE.

### Letter from Dr. T. J. Tyner, of Austin, Representative of Texas State Medical Association to the British Medical Society, at Glasgow.

GLASGOW, SCOTLAND, August 11, 1888.

*Editor Daniel's Texas Medical Journal:*

The great British Medical Association adjourned yesterday. It is very generally conceeded to have been one of the most successful meetings ever held. (Did you ever know one, in any country, which was not so?) I would not like to seem prejudiced, or invidious, but feel justified in saying, our American Medical Association, in character of men, and work done, compares favorably with this. The meeting was held in the University building. Some of the incidents of its birth and life are very interesting. "The foundation was due to Wm. Turnbull, Bishop of Glasgow, who, with the approval of King James II, applied for, and obtained from Pope Nicholas V, a Bull instituting in Glasgow a University, or 'Studium Generale' in Theology, Common and Civil Law, and every other lawful faculty. This charter was granted on the 7th of January, 1450, and after taking into account the salubrity of the climate, and the general suitability of Glasgow as a center of learning, it ordained that the doctors, masters, graduates and alumni should have like immunities and privileges to those enjoyed by the members of the University of Bologna, at that time one of the most celebrated of the Continental schools."

Although begun under such favorable auspices, and taking rank with the foremost school in the world, success was doubtful, and in the earlier half of the sixteenth century, owing to disturbances leading up to the Reformation, the University was reduced to the lowest ebb, and when in 1560, by an act of Parliament, the reformed church of Scotland was established, the priests of the church of Rome were deprived of their livings, and the archbishop fled to France, carrying with him all the more important documents, the mace and insignia of the University. The mace was not restored until 1590. Queen Mary expresses the state of affairs in the following quaint words :

"Forasmekil as within the citie of Glasgow ane college and universitie was devisit to be, quhairin (wherein) the youthe mycht be brocht up in letres and knawlege, the commoune welth servit (served) and virtue incressit—of the quhilk (said) college ane parte of the schulis and chalmeris being bigrit (built), the rest thairof, alswirl duellings (dwellings) as provisioune for the pouir bursoris and masters to teche ceissit (ceased), suer (so) that the samyn aperit (appeared) rather to be the decay of ane universitie, nor ony wyse to be reknit ane establisssit fundation."

To remedy this condition, Queen Mary founded five bursaries for poor students, and presented to the University the buildings of the Black Friars, with thirteen acres of land.

The chair of Medicine was founded in 1637, but five years later it was decided, "Anent the professioun of medicine, the visitation finds that professioun is not necessar for the College in all tyme coming, but withal finds it just that Mr. Robert Maym, who is alreadie in that professioun, continue in the same during his tyme."

The present buildings, which were opened in 1870-1, cost over a million and a quarter dollars. The institution is built on the most modern plan, and combines with beauty of architecture and site, every convenience.

The President's address, W. T. Gairdner, M. D., LL. D., on "The Physician as Naturalist," showed great research and profound thought, but is so lengthy I shall not attempt an analysis. *The* paper of the occasion, by all odds, was by Dr. MacEwing, on "Localization of Brain Lesions." At its conclusion, the feeble, insipid "hear, hear" was inadequate in expression, and the vast audience absolutely (with all their decorum) rose, and gave a regular old Kentucky yell.

The work in sections (of which there were twelve) appears eminently satisfactory. I attended only the section on Ophthalmology, and, of course, in justice to the others, will confine myself to the proceedings in that section. On Wednesday—the first day the sections were opened—Mr. Brunell Carter opened special discussion on the treatment of senile cataract. Drs. Wolfe, Prichard, Teel, Powers and others participated. Dr. Wolfe holds on to the iridectomy and always makes it downward, and also preliminary to the final extraction, if possible. Prichard warmly supports the iridectomy, and also makes it preliminary. In immature cataract he follows Forster, and triturates the lens freely, keeping the arterior chamber free of aqueous. He claims that there is but little or no danger, and the cataract matures in from two to four weeks. He and Wolfe both frequently extract six or eight days after a preliminary iridectomy. Dr. Powers has given up the iridectomy, except when there are adhesions, using a narrow Beer's knife. When the counter puncture is made, the knife is turned directly outward, thus finishing the section midway between the meridian and upper edge of cernea. The advantage is, he claims, that prolapse of the iris is almost impossible, and should it occur, it could only be very slight and easier to reduce. Except one or two questions there was no discussion following his paper. As for myself, I was not convinced that it was either safe or practical. As it is with American surgeons, so it is here, and also in France and Germany, the question as to the iridectomy in cataract extraction is far from settled, though the simple method seems to be gaining ground, and the method given to us by Von Græfe, and which was universally adopted, and practised for so many years, is hanging in the balance, with the probability of being laid aside, and for the very method it absolutely supplanted.

Thursday Dr. H. B. Heniston read a paper on general neurosis, having ophthalmic origin. Nothing specially new was presented. However, in the discussion, Dr. T. H. Bickerton stated that Dr. Carter was the first English surgeon who called attention to this, particularly in relation to epilepsy, but said, Americans, as in many other things, were ahead by several years; to which I *smiled* my thanks.

This gentleman read a paper on "Sailors and their eyesight," in which he made a strong plea for the examination of the vision of seamen.

Mr. Brunell Carter gave the most interesting paper of the day: "The operation of opening the sheath of the optic nerve for the relief of pressure."

The very ingenious instruments for the operation were shown to the section. He took the position that in many cases of optico-neuritis, so-called, the trouble was an accumulation of fluid in the sheath. The idea originated with D. Wecker, and the operation was also done once or twice by him.

Dr. Carter proceeds thus: The external rectus is freely divided, and the eye rotated inwards as far as possible, when a flat-like hook is made to glide around the nerve; it is then brought into full view and grasped with a fine hook, the sheath carefully opened, in which a probe-pointed bistoury is introduced, and the sheath is split as far back as possible. The fluid escapes and relief is immediate, and sight, if not entirely lost, is restored complete; or at least in part.

Friday (last day) the section was engaged with the following subjects: "Treatment of Entropion and Trichiasis, by the transplantation of buchal mucous membrane." "Treatment of Sympleperon by transplanting mucus membrane from the lip." "Chronic nasal catarrh as a reflex cause of accommodation asthenopia." "Iridectomy, schletomy and parencitis of vitreous in chronic glaucoma." They were all good papers, but there was nothing new or worthy special comment. In the afternoon Dr. Wolfe operated at the ophthalmic infirmary for symblepheron by transplantation of Rabbit's conjuctiva. I thought him a little shaky and *a bit* awkward, but when the operation was finished it showed the work of an artist.

On Tuesday this gentleman made twelve cataract extractions, all within two hours, and to-day they are progressing favorably. I suppose he had them rounded up for the occasion.

The social features were delightful, and smoothly carried out. Picnics, garden parties, "conversaciones" and excursions. These were given by the local medical association, the Lord Provo and the Magistrates. The excursions took place to-day (Saturday.) There were eight, each one made up of from 100 to 250, and were so arranged that each one was through some historic and classic spot, made so by Sir Walter Scott, Bobbie Burns, Sir William Wallace, etc. : Falls of the Clyde, ruins of Craignathen Castle, the scenes of Scott's "Old Mortality," traditional birthplace of St. Patrick, Lanark, the county town, in which Glasgow is situated and around which cluster some of the most beautiful traditions of Scotland's folk lore, and the place where Wallace struck his first blow for the freedom of Scottland, by killing the English sheriff in his own office. The Scotch character is a very loveable one, and for pure and sincere hospitality they are scarcely surpassed. To that characteristic cordiality I am indebted to Dr. J. R. Granger for one of the most interesting and pleasant days. I think he would make a splendid|American.

